# *Asteriscus graveolens* Extract in Combination with Cisplatin/Etoposide/Doxorubicin Suppresses Lymphoma Cell Growth through Induction of Caspase-3 Dependent Apoptosis

**DOI:** 10.3390/ijms19082219

**Published:** 2018-07-30

**Authors:** Zainab Tayeh, Rivka Ofir

**Affiliations:** 1 Dead Sea & Arava Science Center, Sapir 868215, Israel; zainab_tayeh@yahoo.com; 2 French Assoc. Inst. for Agriculture and Biotechnology of Drylands, Jacob Blaustein Institutes for Desert Research, Ben-Gurion University of the Negev, Midreshet Ben-Gurion 8499000, Israel; 3 Regenerative Medicine &Stem Cell Research Center, Ben-Gurion University of the Negev, Beer-Sheva 84105, Israel

**Keywords:** cisplatin, cancer, ROS, combinatorial therapy, toxicity, cell death, Topoisomerase I, apoptosis

## Abstract

Chemotherapy drugs action against cancer is not selective, lead to adverse reactions and drug resistance. Combination therapies have proven more effective in defeating cancers. We hypothesize that plant extract/fraction contains many/several compounds and as such can target multiple pathways as cytotoxic agent and may also have chemo sensitizing activities. We designed a study in which, *Asteriscus graveolens* (*Forssk.*) *Less* (*A. graveolens*)-derived fraction that contains sesquiterpene lactone asteriscunolide isomers (AS) will be tested in combination with known chemotherapy drugs. Successful combination will permit to reduce chemotherapy drugs concentration and still get the same impact on cancer cells. Sesquiterpene lactone such as asteriscunolide isomers is a naturally occurring compound found in a variety of fruits, vegetables, and medicinal plants with anti-cancer properties. The experiments presented here showed that adding plant fraction containing AS permit reducing the concentration of cisplatin/etoposide/doxorubicin in order to reduce mouse BS-24-1 lymphoma cells (BS-24-1 cells) survival. It involved enhancing the production of Reactive Oxygen Species (ROS), activation of caspase-3 and inhibition of Topoisomerase I activity. Taken together, the results suggest that *A. graveolens* fraction sensitized BS-24-1 cells to cisplatin/etoposide/doxorubicin through induction of ROS and caspase-3-dependent apoptosis.

## 1. Introduction

Cancer cells main characterization is unlimited proliferation [1]. Aberrant apoptosis may explain why cells that should die survive and lead to cancer progression [2]. Anti-cancer drugs are often accompanied by adverse reactions [3] and may become ineffective due to drug resistance [4] as usually chemotherapeutic drugs target a single pathway. This is the reason drug cocktails are becoming a better option for treating cancer cells [5].

In recent years, the focus in the area of anti-cancer drug development is to elucidate the mechanisms underlying cancer in order to develop target specific drugs [6]. Prior to focusing on the mechanisms of cancer development, chemotherapeutic agents were discovered empirically and were based on inhibiting metabolic pathways necessary for cell division (e.g., DNA damage). However, these anti-cancer strategies were not particularly specific for cancer [7,8] and chemotherapeutic agents affect rapidly growing normal cells as well as cancer cells [9]. Overall, a better understanding of the molecular mechanisms behind the modulation of cellular responses needs to be developed in order to optimize new therapeutic strategies for the use of chemotherapies [10]. Cell death occurs upon simultaneous activation of several signaling pathways, while the specific pathways depend on the type of cancer [11,12,13]. Chemotherapy with a defined dosage used to trigger cancer cell cytotoxicity and sometimes acts through apoptosis activation [14]. Different chemotherapies drugs have different mechanisms of action. Cisplatin can induce DNA damage and reactive oxygen species (ROS) that trigger apoptosis [15]. Cisplatin trigger apoptosis through potential two distinct pathways: one involves the tumor-suppressor protein p53, the other is mediated by the p53-related protein p73 [11]. Doxorubicin and etoposide can induce cell death through several mechanisms such as inhibition of DNA and RNA synthesis, production of free radicals, inhibition of Topoisomerase II and apoptotic response as result of failure to repair DNA Double Strand Breaks (DSB) [16]. Topoisomerase I serves as a target for chemotherapy [17] and its levels can serve as measure for successful chemotherapy treatment in lymphoma models [18].

Currently, drug combinations have been widely used and become the leading choice for treating many diseases, such as cancer. The most important features of any useful drug are: (i) high efficacy and (ii) low toxicity for normal cells. With drug combinations, the hope is that the drugs will have synergistic additive effects in killing cancer cells, while producing no additional side effects [19]. Synthetic compounds are potential candidates as anti-cancer agents with clinical benefit for several types of solid tumors; however, the efficiencies of these compounds are often accompanied by toxic side effects on healthy tissues and tumor resistance, which in turn leads to secondary malignancies [11]. Cisplatin for example, is a well-known chemotherapeutic drug, and used for treating human bladder, lung, ovarian, head and neck, and testicular cancers [20], but is also known for its cytotoxic side effects in healthy tissues [21]. The focus in developing new drugs for cancer is on synthesis of molecules that will target proteins/enzymes and pathways critical for cytotoxicity and/or apoptosis [16,22].

Animal models as well as cellular models serve as tools to identify combination of less cytotoxic drugs [23]. Combination studies have attempted not only to increase effectiveness, but also lower side effects due to toxicity which may occur at therapeutic levels [9]. Recently, synergistic interactions were identified, for example, between beta carotene and alpha-tocopherol in an in vivo hamster cheek pouch carcinogenesis model [24] and between beta carotene and anti-cancer alkylating agents in vitro with human tongue squamous carcinoma cells [25]. Compounds synthesized to mimic natural entities like sesquiterpenes and flavonoids were effective against various kinds of cancer [26,27] and may be used in combination with chemotherapy drugs [28,29]. It is suggested that natural product can be included in combination with chemotherapeutic drugs in order to reduce toxic side effects and drug resistance. We decided to assess this possibility with *Asteriscus graveolens* (*Forssk.*) *Less* (*A. graveolens*). *A. graveolens*, a medicinal plant located in extreme desert environments, contains in its essential oil *cis*-chrysanthenyl acetate, myrtenyl acetate and kessane [30] and monoterpens and sesquiterpenes including oxygenated sesquiterpenes (6-oxocyclonerolidol and 6-hydroxycyclonerolidol) [31]. *A. graveolens* exhibit antimicrobial activity [32] and anti-fungal activity [33]. We studied its anti- cancer activity [34]. Here we describe the study with *A. graveolens* ethyl acetate crude extract-derived fraction following fractionation in methyl *tert*-butyl ether. The fraction, termed here 122.4, contains 4-Hydroxy-2-methylacetophenone, Syringaldehyde, Oplopanone and three major picks of asteriscunolide isomers A–D. Recently, it has been shown that naturally occurring asteriscunolide A induces apoptosis [35].

The purpose of this study is to describe the molecular mechanisms that mediate the sensitivity of cancer cells to combined treatment of cisplatin/etoposide/doxorubicin with *A. graveolens*-derived fraction 122.4. We showed that fraction 122.4 that induces cytotoxic effects against mouse lymphoma cells (BS-24-1 cells) in a selective manner (marginal toxicity to non-cancerous cells), acts in synergistic manners with these known chemotherapeutic agents. Such synergisms enable to reduce the amount of cisplatin/etoposide/doxorubicin required to kill cancer cells and may lead to fewer side effects.

## 2. Results and Discussion

### 2.1. A. graveolens-Derived Fraction 122.4 in Combination with Cisplatin/Etopside/Doxirobucin Reduces BS-24-1 Cell Viability

**Figure 1:**
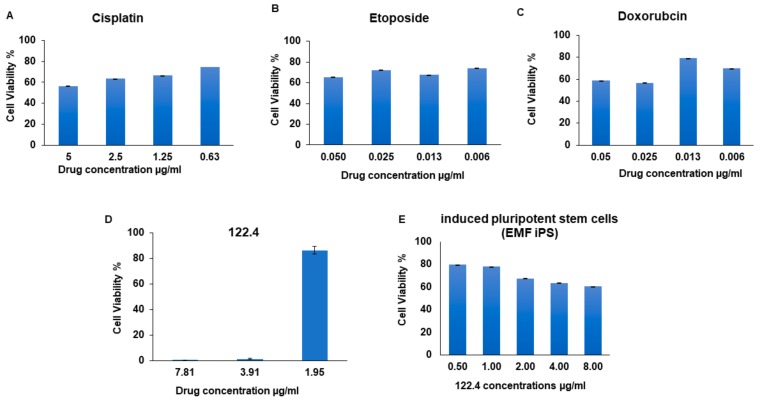
The effects of treatment with *A. graveolens fraction* 122.4 or several chemotherapeutic drugs on BS-24-1 cells and human induced pluripotent stem cells (iPSCs). A–C, cytotoxic effects of cisplatin, etoposide and doxorubicin on BS-24-1 cells, respectively. D and E, cytotoxic effects of 122.4 on BS-24-1 cells and iPSCs, respectively. iPSCs cells were plated at a concentration of 500,000 cells/mL and incubated with fraction 122.4 for 72 h; the results are presented as the means ± SD and are representative of three independent experiments.

In this project we assessed whether natural products can synergized with chemotherapeutic drugs without reducing cytotoxicity to cancer cells. BS-24-1 cells (mice T lymphoma [36]) served as cancer model in vitro and *A. graveolens*-derived fraction, termed here 122.4, is the nature derived product. The cytotoxic effects and the mechanism of action of *A. graveolens* crude extracts and fractions were previously described [34]. The cytotoxic effect of cisplatin, etoposide and doxorubicin on BS-24-1 cells is presented in Figure 1A–C, respectively. 122.4 acts in selective manner as the dose that kills 80% of BS-24-1 cells is a four-fold lower than the dose required to kill non-cancerous cells (Figure 1D,E, respectively). Human induced pluripotent stem cells serve as non-cancerous control cells; they were generated by reprogramming fibroblasts originated from a skin biopsy of a healthy person as previously reported [37].

The concentration of 122.4, chemotherapeutic agents (cisplatin, etoposide, doxorubicin) needed to kill 10–90% of BS-24-1 cells (Inhibitory Concentration, IC10–IC90) are displayed in Table 1. To characterize the interactions between 122.4 and cisplatin, etoposide, doxorubicin, data were analyzed using the median-effect method of Chou using combosyn program [38]. The method is based on generating dose-response curves for each agent individually in the combination of two agents tested, and analyzing the results obtained from the combination treatment within the same experiment (as described in Materials and Methods). Experiments with different concentrations of 122.4 and cisplatin, etoposide, doxorubicin, were performed in order find the combination that lead to maximum death of BS-24-1 cells. Finally, the concentration of IC5, IC80 and IC10 for cisplatin, etoposide, doxorubicin, respectively (fixed concentration) were combined with different concentrations of fraction 122.4 (non-fixed concentration) in the range of (0.64–0.93 μg/mL). Cells were incubated for 72 h in the combined protocol and under these conditions, 0.64 μg/mL of 122.4 lead to 99% cell death (IC99; Table 2).

**Figure 2:**
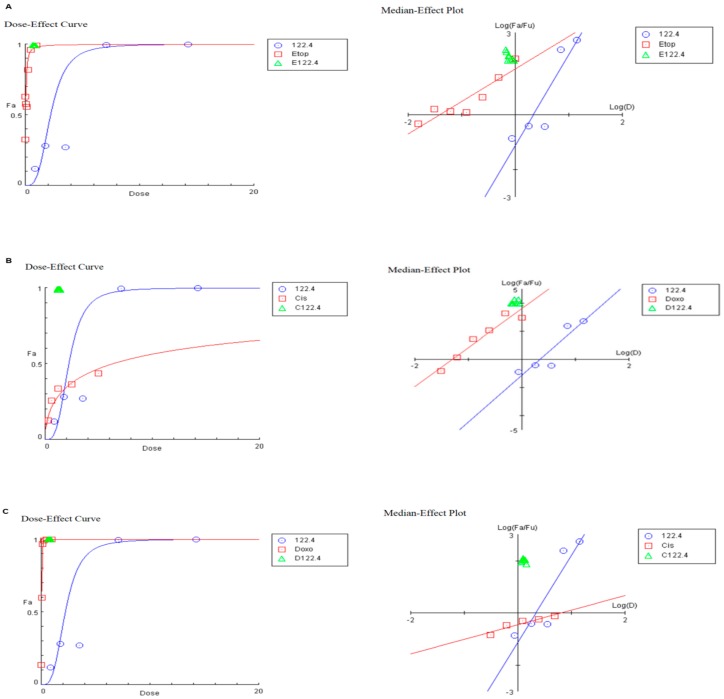
Dose-Effect Curve and its linearization with the Median-Effect Plot for a single and combination treatment of 122.4 and chemotherapy drugs. Right side represents the median-effect plot of single/combination treatment and the left side shows the dose-effect curve of 122.4 with etoposide (Etho), cisplatin (Cis), doxorubicin (Doxo), **A**–**C**, respectively. The red line represent chemotherapeutic single treatment, blue line represent the single treatment of 122.4 and green triangle is the combination treatment using ComboSyn (E122.4, C122.4, D122.4 are combinations of 122.4 with etoposide, cisplatin and doxorubicin, respectively).

Combination of anti-cancer drugs may produce cytotoxic effects greater than or less than the predicted individual cytotoxicity. Creating curves of constant effect (isobole), enables to suggest synergistic cytotoxicity. Graphic illustrations as isobolograms describing synergic action of 122.4 and etoposide/cisplatin/doxorubicin are shown in Figure 2A–C, respectively in which the drug combinations dose-response curves of BS-24-1 cells with 122.4 are plotted using ComboSyn (http://www.combosyn.com). As shown in Figure 2 and listed in Table 3, BS-24-1 cells have the shape of the dose-effect curves, which are depicted by the m value in Table 3 (m = 1, >1 and <1 indicates hyperbolic, sigmoidal and negative sigmoidal curve, respectively). We need both Potency (Dm) and Shape (m) to determine the drug synergism.

Since a larger m value indicates a steeper dose-effect curve, 122.4, doxorubicin and etoposide have increase dose-effect curves (higher m values) while cisplatin has shallow dose-effect curves (lower m values) and as such will be less responsive to drug combinations in BS-24-1 cells.

This kind of analysis can serve as a platform to check the right combinations of drugs and natural derived materials in relation to a desired mechanism of action and relevant to each kind of cancer.

### 2.2. Mechanisms of Action of Treatment by Two-Drug Combinations

The effects of treatment by two-drug combinations of 122.4 with either of the chemotherapeutic cisplatin, etoposide, and doxorubicin were assessed. Several mechanisms involved in cell death were studied: inhibition of Topoisomerase I (Topo I), activation of apoptosis (caspase-3) and production of ROS.

**Figure 3:**
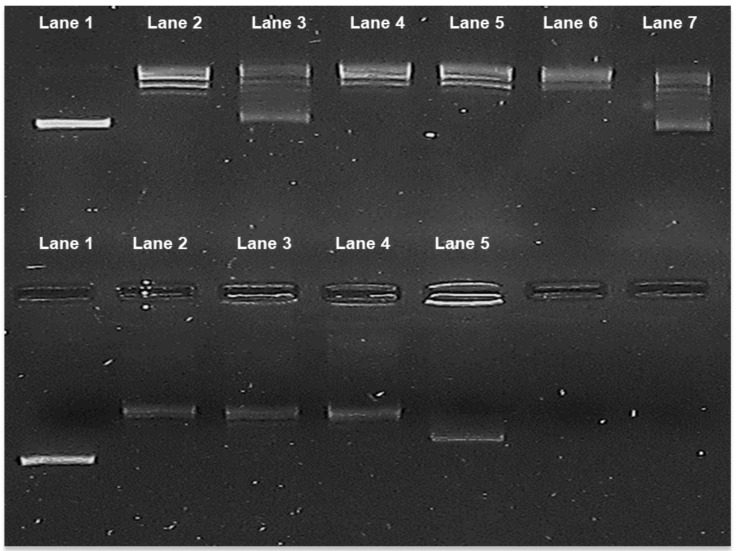
Inhibition of Topoisomerase I activity by single or two-drug combinations treatment. Purified Topo I enzyme from calf thymus was assessed. **Upper part**: Lane 1, supercoiled untreated plasmid pUC19, Lane 2, plasmid + Topo I (Relaxed form), Lane 3 and 4: treatment with 122.4, 800 μg/mL and 27 μg/mL, respectively. Lane 5, 6, 7 treatment with cisplatin (80 μg/mL), etoposide (1600 μg/mL) and doxorubicin (160 μg/mL), respectively. **Lower part**, Lane 1, supercoiled untreated plasmid pUC19, Lane 2, plasmid + Topo I (relaxed form), Lane 3, 4 and 5: combination treatment of 122.4 (0.64 μg/mL) and cisplatin (0.54 μg/mL), etoposide (0.03 μg/mL) and doxorubicin (0.0078 μg/mL), respectively. For the combination treatment the concentrations of IC99 in Table 2 were used. We further studied whether combining 122.4 with cisplatin, etoposide, and doxorubicin will permit reducing the concentrations needed for the activation of caspase-3 by cisplatin, etoposide, and doxorubicin. Following incubation of 3 × 10^6^ cells/mL overnight with 100 μg/mL of doxorubicin/etoposide/cisplatin, five-fold increases in caspase-3 activity was observed (Figure 4). Adding 122.4 enabled to reduce the concentration of doxorubicin or etoposide or cisplatin to 50 μg/mL and still reach similar caspase-3 activation (Figure 4). In order to assure that caspase-3 is the induced enzyme, pre-incubation with caspase-3 inhibitor Ac-DEVD-CHO was performed. The results imply that compound (s) within 122.4 sensitize BS-24-1 cells in such a way that doxorubicin or etoposide or cisplatin became a better activators of caspase-3. If apoptosis is considered to be an optional mechanism to kill tumor cells, it will be worthwhile to think of combining these chemotherapeutic drugs with 122.4 content.

We tested the effect of adding 122.4, cisplatin, etoposide, and doxorubicin alone or the combinations of 122.4 with each of cisplatin, etoposide, and doxorubicin on the activity of purified Topo I enzyme (Calf thymus, Takara, Kusatsu, Japan). Active Topo I change the conformation of pUC19 supercoiled DNA plasmid into relaxed form. As seen in Figure 3, treatment with 122.4 or doxorubicin alone but not with cisplatin or etoposide leads to Topo I inhibition; ~50% of pUC19 remained as supercoiled. Combined treatment of 122.4 with doxorubicin induced a stronger Topo I inhibition as compared to 122.4 alone or doxorubicin alone (in the combined treatment the pUC19 plasmid basically remained supercoiled). Adding etoposide or cisplatin to 122.4, interferes with Topo I inhibition by 122.4, suggesting that these drugs antagonized the Topo I inhibitory activity of compound(s) in 122.4.

The results imply that when inhibition of Topo I enzyme is considered to be the target for treatment, 122.4+doxorubicin are good combination while 122.4+ etoposide or 122.4+ cisplatin are bad combinations.

**Figure 5:**
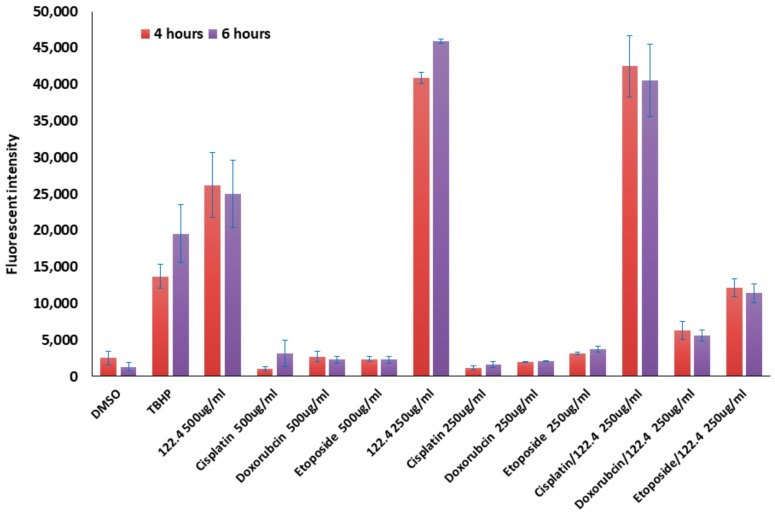
The effect of 122.4 on ROS production in BS-24-1 in combined treatments. 5 × 10^5^ cells/well were treated with 250–500 μg/mL in a single treatment assay and with 250 μg/mL of both 122.4 and chemotherapeutic drugs in the combined treatment assay. *X*-axis represents the different treatments. Y-axis represents fluorescence intensity (ROS levels).

Agents that increase the levels of ROS in cancer cells lead to cytotoxicity to these cells [39,40]. We assessed the levels of ROS when 122.4 was combined with doxorubicin or etoposide or cisplatin in treating BS-24-1 cells. 500,000 cells/well were stained with 20 μM of 7′-dichlorodihydrofluorescein diacetate (H2DCFDA) for 30 min and then 250–500 μg/mL doxorubicin or etoposide or cisplatin was added to the cells in the single treatment experiment. 250 μg/mL of doxorubicin or etoposide or cisplatin were combined with 250 μg/mL of 122.4 were added to the cells in the drug combination experiments. ROS signal was detected following 4 and 6 h. As seen in Figure 5, low levels of ROS were detected in single treatment experiments with doxorubicin or etoposide or cisplatin (drug concentration 250 and 500 μg/mL). However, the level of ROS increased from 2–30 folds when 122.4 (250 μg/mL) was added to doxorubicin or etoposide or cisplatin (250 μg/mL). The higher effect was monitored when 122.4 was combined with cisplatin.

These results strongly imply how important it is to use the combined treatment when the mechanism to kill specific cancer cells involves ROS generation. High levels of ROS contribute to cancer cell death; apparently, doxorubicin or etoposide or cisplatin alone are not efficient in elevating ROS levels but with 122.4 they became more potent in ROS generation.

*Asteriscus graveolens* (*Forssk.*) *Less* (*A. graveolens*)-derived fraction 122.4 contains sesquiterpene lactone asteriscunolide isomers (AS). It is interesting to note that sesquiterpene lactone is a group of compounds with versatile structural components that react with functional groups in proteins and enzymes and are considered for anti-cancer drug development [41,42]. The results suggest that the compounds within 122.4, including those belong to the group of sesquiterpene lactone, are in charge of improving the activity of known chemotherapeutic drug like doxorubicin/etoposide/cisplatin in combined treatments. We think that treating cancer cells with combined agents: plant extracts (that usually contain various compounds) and chemotherapeutic drugs will activate several pathways that lead to cell death like apoptosis, ROS generation, inhibition of Topoisomerase I and maybe more pathways, and as such will be a more efficient anti-cancer therapy.

## 3. Material and Methods

### 3.1. Reagents

Doxorubicin, etoposide and cisplatin were obtained from Sigma-Aldrich, Rehovot, Israel. All reagents were dissolved in dimethyl sulfoxide (DMSO, Sigma-Aldrich, Rehovot, Israel)) and stored frozen as 1, 2, 10, 20 mg/mL stock solution in DMSO and diluted as needed. We used the following kits: Reactive oxygen species kit (Abcam, Cambridge, UK), Caspase-3 kit (Bio-vision Inc., Milpitas, CA, USA), Topoisomerase I (Takara), DNA plasmid pUC19 (Thermo scientific Inc., Waltham, MA, USA).

### 3.2. Cells

Mouse lymphoma cell line (BS-24-1 cells, [43]) used for these studies was cultured at 37 °C with 5% CO_2_ in RPMI-1640 medium with 20% fetal bovine serum (FBS) and penicillin/streptomycin (BI Biological Industries, Beit Haemek, Israel). Human induced pluripotent cell line (iPSCs, generated by reprogramming embryo foreskin fibroblast and termed EMF) were cultured as previously described [37].

### 3.3. Isolation of A. graveolens-Derived Fraction 122.4

Ethyl acetate extract prepared from dry leaves of *A. graveolens* was dried using a Turbo Vaporator (40 °C) to yield crude extract as previously described [34]. The crude extract was dissolved in Methyl *tert*-butyl ether (MTBE), mixed with silica gel and following solvent evaporation, loaded on a glass column (silica gel 60, 75 g, 0.04–0.063 nm, 10 × 04.5 cm). The column was eluted with the following solvents (each solvent was collected separately): hexane, hexane/petroleum ether (1/1), petroleum ether, petroleum ether/MTBE (1/1), MTBE, MTBE/dichloromethane (1/1), dichloromethane, chloroform, acetone, acetonitrile and methanol. The fractions were evaporated in the hood overnight. Chromatography on silica columns was repeated for fractions that exhibited biological activity until GC-MS showed that the active fraction contained also sesquiterpene lactone asteriscunolide isomers (AS). Stock solutions of 10 mg/mL of fractions containing AS (termed here 122.4) was dissolved in DMSO and aliquots were frozen at −20 °C. The following compounds were identified in fraction 122.4: 4-Hydroxy-2-methylacetophenone, Syringaldehyde, plopanone and three picks of asteriscunolide isomers (we could not define exactly which of the known A–D isomers).

### 3.4. Cytotoxicity Assays

Cytotoxicity was assessed using a dimethyl-thiazol diphenyl tetrazolium bromide (MTT) assay. MTT (AppliChem GmbH, Darmstadt, Germany) assay was performed as previously described [43]. In MTT assay, the metabolic activity of the cells is monitored. It is a convenient colorimetric assay that measure the activity of Nicotinamide adenine dinucleotide phosphate (NAD(P)H)-dependent cellular oxidoreductase enzymes. There is a correlation between the metabolic activity and the number of viable cells. Cells were treated with 122.4 and different chemotherapeutics drugs for 72 h. Following incubation for 2 h with MTT reagent, plates were read on wavelength 570 nm.

### 3.5. Topoisomerase I Assay

Inhibition of Topoisomerase I (Topo I) activity by plant extracts was performed with Topo I purified enzyme (Calf thymus, Takara, Japan). Fraction 122.4 and different chemotherapeutic drugs were added to Topo I-specific reaction mixture [20 mM Tris-HCl (pH 8.0), 1 mM dithiothreitol, 20 mM KCl, 10 mM MgCl_2_, 1 mM EDTA, 30 μg/mL bovine serum albumin and 225 ng pUC19 supercoiled DNA plasmid] in a final volume of 25 μL, before addition of Topo I. The reaction was terminated by adding 5 μL of stop buffer (final concentration: 1% SDS, 15% glycerol, 0.5% bromophenol blue, and 50 mM EDTA pH 8). The reaction products were analyzed by electrophoresis on a 1% agarose gel with Tris/Borate/EDTA(TBE) buffer (89 mM Tris-HCl, 89 mM boric acid, and 62 mM EDTA) and the gels were photographed using a short wavelength UV lamp (Chemilmager^TM^ 5500 equipment, Alpha Inotech Corporation, San Leandro, CA, USA).

### 3.6. Reactive Oxygen Species

Intracellular ROS production was monitored by the permeable fluorescence dye, 2′, and 7′-dichlorodihydrofluorescein diacetate (H2DCFDA). Interaction between H2DCFDA and ROS form the fluorescent product 2,7-dichlorofluorescein (DCF). The intracellular fluorescence intensity of DCF is proportional to the amount of ROS generated by the cells. 1.5 × 10^5^ BS-24-1 cells were incubated with 20–25 μM of H2DCFDA for 30 min at 37 °C, under 5% CO_2_ followed by the addition of different concentrations of 122.4 and of the chemotherapeutic drugs. The fluorescence intensity of intracellular DCF (excitation 485 nm, emission 535 nm) was measured using a spectrofluorometer (TECAN, SPECTRAFLUOR PLUS, Tecan Group Ltd. Männedorf, Switzerland). 50 μM of the ROS-inducer, Tertiary butyl hydroperoxide (TBHP), was used as a positive control.

### 3.7. Caspase-3 Activity Assay

Cells were treated with 122.4 and different chemotherapeutic drugs for 4 to 8 h. Cells were observed under the microscope for morphological changes associated with the beginning of apoptosis. As soon as apoptosis began, cells were centrifuged at 13,000 rpm at 22 °C and washed three times with PBS (137 mM NaCl, 2.7 mM KCl, 10 mM Na_2_HPO_4_, 2 mM KH_2_PO_4_, pH 7.4). Cells were re-suspended in 50 μL of chilled cell lysis buffer and incubated on ice for 10 min followed by 1 min centrifugation at 10,000 rpm. Cell extracts equivalent to 50 μg protein were analyzed for caspase-3 activity using a Caspase 3 Assay Kit (Bio-vision) according to the manufacturer’s instructions. To validate that the activity observed was caspase-3, 50 μg cellular extract was pre-incubated with 10 μL of the caspase-3 inhibitor solution (50 mM), Ac-DEVD-CHO, for 10 min before the enzymatic reaction start by addition of the caspase-3 substrate.

### 3.8. Median-Effect Analysis

To characterize the interactions between 122.4 and chemotherapeutic drugs, data were analyzed by the median-effect method of Chou using the ComboSyn program [38]. In this method, for every combination of 2 agents tested, dose-response curves are generated for each agent individually, and these data are used to analyze the results obtained from the combination treatment within the same experiment. A commonly used approach is to prepare a stock medium containing the 2 agents in which the first concentration used is the concentration required to inhibit 20% of the cell culture. The concentration of IC20 of all chemotherapy agents were combined with different concentration of the plant extract 122.4, in order to test the drugs over a range of doses of 122.4 with the lowest dose of chemotherapies. Therefore, we focused our efforts on studying the effects of combining a varying dose of 122.4 with lowest fixed doses of chemotherapy. The range of (0.64–0.93 μg/mL) of 122.4 concentrations was used to generate the median-effect analysis plots. A combination index (CI) number was generated using a commercially available software program ComboSyn. For each experiment, linear regression analysis of the dose-response data was used to generate a median-effect value Dm (corresponding to the IC50) along with a slope m and linearity coefficient r. For all data reported here, r values were more than 0.91. The CI is calculated using the formula: CI = (D)1/(Dm)1 + (D)2/(Dm)2/(D)1(D)2/(Dm)1(Dm)2. This applies to 2 mutually nonexclusive drugs with independent modes of action, where (D)1 and (D)2, for example, represent 122.4 and a chemotherapeutic agents, respectively, and (Dm) refers to the dose of each drug that, by itself, will generate a median effect (i.e., the IC50) from the data within that experiment. As indicated, we used non-fixed ratio of the 122.4 drug and a fixed value of chemotherapies drugs to better approximate what might occur in a patient treated with continuous 122.4. Although quantifying the degree of synergy or antagonism is of uncertain validity, this method of analysis generally defines CI values of 0.9 to 1.1 as implying additivity, 0.3 to 0.9 as synergistic, less than 0.3 as strongly synergistic, 1.1 to 3.3 as antagonistic, and more than 3.3 as strongly antagonistic [38].

## 4. Conclusions

We report here that *Asteriscus graveolens*-derived fraction 122.4 that contains 4-Hydroxy-2-methylacetophenone, Syringaldehyde, Oplopanone and three major picks of asteriscunolide isomers A-D, can induce cytotoxic effects against mouse lymphoma cells (BS-24-1 cells) in synergistic manner with chemotherapeutic agents.

We show that combination with 122.4 enabled to use 50% lower dose of cisplatin, doxorubicin and etoposide and still get similar levels of caspase-3 activation. Combination with 122.4 results in higher ROS production comparing to treatment with cisplatin, doxorubicin or etoposide alone. Combined treatment of 122.4 and doxorubicin, results in better inhibition of Topoisomerase I as compared to single treatments.

We suggest that fraction 122.4 contains various metabolites that in two-drug combinations with chemotherapeutic agents react in synergism with cisplatin or doxorubicin or etoposide to activate multiple pathways that are involved in cell death mechanisms. We see advantage in including plant extract in combined cancer therapy; it has the potential to attack cancer cells in more than one mechanism and as such may be more effective to cancer patients.

## Figures and Tables

**Figure 4:**
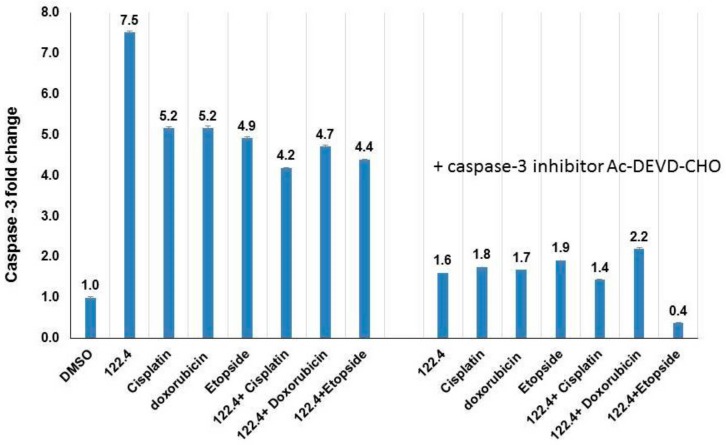
Induction of Caspase-3 activity in BS-24-1 cells with single and combined treatments. 3 × 10^6^ cells/mL were treated overnight with 100 μg/mL of 122.4 or doxorubicin or etoposide or cisplatin as single treatment or with 50 μg/mL each as combined treatment. In order to assure that caspase-3 is the induced enzyme, cellular extracts were pre-incubated with caspase-3 inhibitor Ac-DEVD-CHO for 10 min before adding the substrate for the caspase-3 assay. *Y* axis presents folds induction as compared to incubation with the solvent DMSO.

**Table 1:** Inhibitory concentrations (IC; IC10–IC90) of single treatments of BS-24-1 cells. IC10–IC90 of single treatment with 122.4 or doxorubicin or etoposide or cisplatin was calculated using Excel. The results presented are representative of three independent experiments.

Treatment	122.4	Doxorubicin	Etoposide	Cisplatin
IC 10	3.236	0.005	0.004	3.264
IC 20	3.555	0.012	0.008	46.545
IC 30	3.875	0.019	0.012	67.549
IC 40	4.194	0.026	0.016	88.553
IC 50	4.514	0.033	0.020	109.557
IC 60	4.833	0.040	0.024	130.561
IC 70	5.152	0.047	0.028	151.565
IC 80	5.472	0.054	0.032	172.569
IC 90	5.791	0.060	0.036	193.573

**Table 2:** The inhibitory concentration 99 (IC99) of drug combinations on BS-24-1 cells. A final concentration of 0.64 µg/mL of 122.4 (equal to IC5 of 122.4) was needed in a combination with the IC5, IC80 and IC10 of cisplatin, etoposide, doxorubicin, respectively. Data shown are pooled results of minimum of three experiments using ComboSyn. IC5 calculation was performed using the linear regression line equation.

**IC99**	**122.4 Etoposide**	**122.4 Cisplatin**	**122.4 Doxorubicin**
	0.64 + 0.03 ± 0.00064	0.64 + 0.54 ± 0.00028	0.64 + 0.0078 ± 0.00113

**Table 3:** Dose-effect relationship parameters for treatment by two-drug combinations of 122.4 with etoposide or cisplatinor doxorubicin. BS-24-1 cells were treated with the two-drug combinations. Potency, shape (sigmoidicity) and conformity of dose-effect curve (linear correlation coefficient) are represented by Dm, m, and r, respectively, where Dm (ED50) is the antilog of x-intercept in µg/mL, m is the slope of the median-effect plot signifying the shape of the dose-effect curve (m = 1, >1 and <1 indicates hyperbolic, sigmoidal and negative sigmoidal curve, respectively), and r is the linear correlation coefficient of the median-effect plot. Data shown are pooled results of minimum of three experiments.

Drug	Dm	M	r
Etoposide	0.0383	1.185	0.919
Cisplatin	6.32533	0.555	0.945
Doxorubicin	0.049	2.754	0.966
